# Exploring the Cerebro-Renovascular Link: Implications of Renal Haemodynamics on Intracranial Arterial Calcification

**DOI:** 10.14336/AD.2025.0489

**Published:** 2026-05-05

**Authors:** Joseph Amihere Ackah, Xiang-yan Chen, Huixing Zeng, Yiming Liu, Youcheng Rong, Xuelong Li, Ben Yuk-fai Fong, Ximin Pan, Feng Zhang, Jing Cai

**Affiliations:** ^1^Department of Health Technology and Informatics, Faculty of Health and Social Sciences, The Hong Kong Polytechnic University, Kowloon, Hong Kong, China.; ^2^Division of Science, Engineering and Health Studies, College of Professional and Continuing Education, The Hong Kong Polytechnic University, Kowloon, Hong Kong SAR, China.; ^3^Department of Ultrasonography and Biomedical Innovation Center, The Sixth Affiliated Hospital, Sun Yat-Sen University, Guangzhou, China.; ^4^Department of Neurology, The Second Affiliated Hospital, Guangzhou Medical University, Guangzhou, China.; ^5^Department of Radiology and Biomedical Innovation Center, The Sixth Affiliated Hospital, Sun Yat-Sen University, Guangzhou, China.

**Keywords:** intracranial arterial calcification, aging-related intracranial atherosclerosis, renal impairment

## Abstract

Evidence hints that the cerebro-renovascular pathway could offer promising approaches to enhancing renal health and reducing the associated risk and burden of intracranial arterial calcification (IAC), a crucial marker for ageing-related intracranial atherosclerosis. This study explored whether renal function and renovascular haemodynamic metrics could predict the severity and load of IAC and elucidate the clinical distinctiveness between intimal and medial IAC. Seventy-seven Chinese participants were enrolled in this cross-sectional study. Kidney functions were evaluated using the estimated glomerular filtration rate (eGFR). Renovascular haemodynamics (on resistance) was assessed using duplex ultrasound to record metrics such as the resistive index (RI) of renal and interlobar arteries. Non-enhanced computed tomography (CT) assessed the count, severity, and load of IAC and classified IAC into intimal and medial. Regression models were fitted for analyses. Among 69 patients with IAC, 29% exhibited predominantly intimal and 71% predominantly medial calcification. Of those with IAC, 26 (37.7%) had an eGFR below 60 ml/min/1.73m², 19 (27.5%) had values between 60-90 ml/min/1.73m², and 24 (34.8%) had scores above 90 ml/min/1.73m². Measures of eGFR <60 ml/min/1.73m² were independently associated with higher renal RI [adjusted OR=3.45 (95%CI: 1.38-8.59, p=.008)]. Patients with predominantly medial IAC had higher renovascular resistance. Higher renal RI independently predicted higher IAC load [adjusted OR=1.88 (95%CI: 1.06-3.35, p=0.032)]. In summary, renovascular haemodynamics significantly determine the load and severity of IAC, particularly in individuals with reduced renal function (eGFR <60 ml/min/1.73m²). The impact of renal impairment is more pronounced on medial IAC than on intimal IAC.

## INTRODUCTION

As a proxy indicator of ageing-related intracranial atherosclerosis, intracranial arterial calcification (IAC) has been independently associated with cerebrovascular accidents and cognitive impairment [1, 2]. IAC is frequently observed on non-enhanced brain computed tomography (CT), which is a routine neuroimaging investigation for patients suspicious of stroke. Our earlier clinical research revealed that approximately 70% of the Chinese population exhibit calcification in cerebral arteries [3], with this prevalence rising to over 80% among individuals who have experienced a stroke [4]. It is shown that IAC is identified in nearly 60% to 90% of internal carotid arteries, up to 30% of vertebral arteries, and approximately 6% of basilar or middle cerebral arteries in a Chinese population [3-5]. Imaging and histopathology-validated investigations distinguished IAC into intimal and medial subtypes, revealing that both may present distinct clinical implications [6-10]. Arterial calcification, particularly in the intimal and medial layers, is a significant pathological process that can deteriorate oxygen and nutrient exchange between blood and tissues [11]. This process is associated with a range of clinical conditions, including cognitive decline, dementia, resistant hypertension, angina, ischaemia of the lower extremities and progressive renal function impairment. Emerging studies highlight the systemic consequences of vascular calcification across multiple organ systems [12, 13]. The evidence demonstrates that calcification of both intimal and medial layers of arteries occurs across multiple vascular beds with clinically significant repercussions.

Clinical studies revealed that IAC independently disrupts cerebral haemodynamics and that higher burdens of IAC lead to generalised increases in blood flow resistance and arterial stiffening in the brain [14, 15]. Subsequently, it was shown that the severity of IAC might be associated with not just the haemodynamics in the cerebral vasculature but also peripheral vasculature [16]. It was demonstrated explicitly that in patients with acute ischaemic stroke, the severity of IAC is positively and independently associated with increased arterial stiffness. This association was observed with peripheral arterial stiffness, measured by brachial-ankle pulse wave velocity (PWV) [16] and with cerebral arterial stiffness, measured by carotid-cerebral PWV [17]. Additional evidence showed that the extensive calcifications in the lower limb, renal, coronary, carotid and cerebral arteries increase with age, diabetes, high systolic blood pressure and those on haemodialysis [13]. These findings further enhanced our understanding of IAC’s clinical significance for both the central and peripheral systemic circulations.

Previous investigations show that changes in renovascular haemodynamic influence systemic circulation through intrarenal autoregulatory processes influenced by myogenic responses, tubuloglomerular feedback, and neurohormonal mediators [18-20]. When compromised, the myogenic response, which regulates vascular tone in response to pressure fluctuations, may result in increased mechanical stress on vascular walls, potentially triggering calcification processes [19, 20]. Additionally, dysregulation of tubuloglomerular feedback can disrupt mineral metabolism, particularly calcium and phosphate balance, which are critical factors in the formation of vascular calcification [21]. Elevated phosphate levels and altered calcium homeostasis, common in chronic kidney disease (CKD), can contribute to higher burdens of calcification in various vascular beds, including the cerebral arteries [22, 23]. Neurohormonal mediators, such as the renin-angiotensin system, further exacerbate this risk by promoting oxidative stress and inflammation, which are known to facilitate vascular calcification. Phosphate retention in severe renal impairment can further increase the propensity for calcification involving intracranial vessels [23, 24]. Understanding the cerebro-renovascular pathway could offer promising approaches to enhancing renal health and reducing the cerebrovascular risks associated with intimal and medial calcifications in patients with impaired renal function [25].

We posited that overlooking the distinct impacts of the different characteristic subtypes of IAC may misleadingly downplay the true association between renal impairment and IAC. This oversight could affect the prognostic evaluation of IAC load, haemodynamics, and management outcomes among patients, as indicated in previous studies [26-28]. Based on the current information, this is the first study to explore the cerebro-renovascular window as a portal to investigate the intimal and medial IAC burdens in renal impairment. We asked the question: could the renovascular haemodynamics coupled with clinical information on renal function predict IAC risks and severity, especially among individuals with reduced renal functions? This study aimed to unravel whether renal function and renovascular haemodynamic metrics could predict the severity and load of IAC and elucidate the clinical distinctiveness between intimal and medial IAC. The findings from this investigation, adjunct to previous investigations, could provide holistic and in-depth information for enhanced therapeutic and management outcomes involving the two distinct patterns of IAC.

## MATERIALS AND METHODS

### Patient selection

This cross-sectional study randomly included 77 participants who underwent both brain CT scans and renal Doppler ultrasound examinations referred to the Sixth Affiliated Hospital of Sun Yat-sen University (Guangzhou, China). The hospital's database identified these participants as the only patients who received both CT and ultrasound examinations as part of their clinical management between January 2021 and December 2023. Inclusion criteria were as follows: (1) age ≥ 18 years; (2) having performed brain CT with 0.6 mm slice thickness as a measurement of calcification; (3) having performed renal artery ultrasound as a measurement of the renovascular blood flow and haemodynamics; (4) estimating estimated glomerular filtration rate (eGFR). Exclusion criteria were: (1) poor CT imaging quality and (2) inadequate clinical data for analysis. The clinical ethics committee of the participating hospital conducted an ethical review of patient inclusion for this study. The institutional review board of the Hong Kong Polytechnic University approved the research protocol [approval ID: HSEARS20240306009]. We adhered to the research protocols under the principles of the Declaration of Helsinki.

### Clinical data collection

All participants' demographic information, including gender, age, body mass index (BMI), current smoking or drinking, and past medical history, including hypertension, hyperlipidaemia, diabetes, stroke, coronary artery disease (CAD), or haemodialysis, as well as medication history, were documented. Moreover, the following clinical information was also obtained: total cholesterol (TC), triglyceride (TG), low-density lipoprotein-cholesterol (LDL-C), high-density lipoprotein -cholesterol (HDL-C), haemoglobin A1c (HbA1c), homocysteine, D-Dimer, fibrinogen, serum creatinine (SCr), systolic blood pressure (SBP), and diastolic blood pressure (DBP). Hypertension was defined as systolic blood pressure ≥140 mmHg, diastolic pressure ≥90 mmHg, or hypertension history. Diabetes was determined as the fasting plasma glucose was ≥7.8 mmol/l, or as HbA1c ≥6.0%, or a medical history of diabetes. Hyperlipidaemia was judged when laboratory examination of the serum showed TC level ≥6.2 mmol/L, TG level ≥2.3 mmol/L, LDL-C level ≥4.1 mmol/L, HDL-C <1 mmol/L, or a medical history of hyperlipidaemia. The eGFR was calculated using the modified glomerular filtration rate estimating equation for Chinese subjects: eGFR (ml/min/1.73m^2^) = 175 × (SCr)^-1.234^ × (age)^-0.179^ × gender coefficient (1 if male or 0.79 if female). The eGFR (ml/min/1.73 m²) of >90, 60-90, and <60 was classified as normal, mildly reduced, and decreased kidney function, respectively.

### CT neuroimaging acquisition

The scan was performed using TOSHIBA Aquilion ONE 640-slice spiral CT. The patient is in a supine position with the head in place and hands naturally placed on either side of the body, and the scanning range covers the aortic arch to the roof of the skull. During the arterial phase, surestart scanning was used, and the descending aorta at the aortic arch level was selected as the monitoring layer, with a trigger threshold of 150-180 Hu. The scanning parameters are as follows: spiral scanning, kV=120, mA=250, 0.5 s/rev, 64 detector rows, layer thickness of 0.5 mm, pitch of 0.828, scanning layer thickness of 0.5 mm, interlayer spacing of 0.5 mm, and recombination layer thickness of 1 mm.

### Assessment of calcification on CT

Two researchers independently evaluated the brain CT images, maintaining excellent interrater reliability (Kappa values >0.9) while being blinded to all clinical data of the patients. The assessments were conducted by an experienced neurologist (X.L.) with over five years of expertise and a trained neuroimaging researcher (J.A.A.) with one year of experience in CT imaging evaluations.

A visual grading method was applied to assess the presence of IAC, which is defined as the hyperdense artery sign with a density of more than 130 Hounsfield units. Seven main intracranial arteries were evaluated, including the C2-C7 segments of the bilateral internal carotid arteries (ICA), the bilateral middle cerebral arteries (MCA), the V4 segments of the bilateral vertebral arteries (VA), and the basilar artery (BA). As previously described [29], the severity of IAC was evaluated by grading values (thickness and extent) for each cerebral artery, and the highest composite CT score of 0-2, 3-5, and 6-8 was respectively classified for IAC severity as mild, moderate, and severe IAC. The total number of arterial segments with calcifications represented the IAC count. The total calcification scores recorded across all arterial segments represented the participant's IAC load.

**Figure 1.**
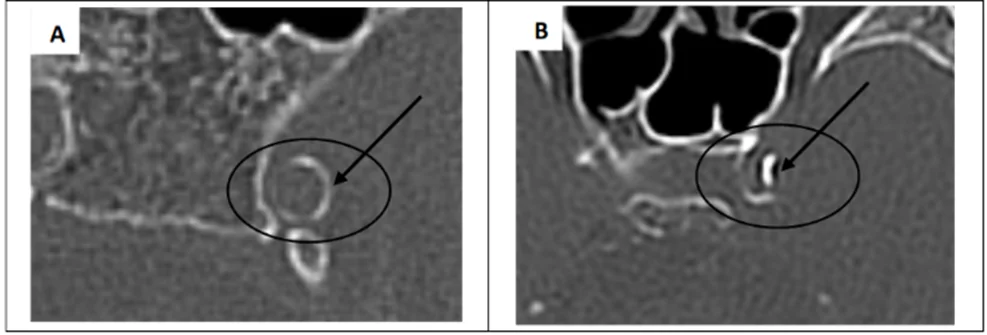
**IAC assessment: classification of IAC subtypes**. (**A**) Calcification spans between 270-360° of arterial circumference, hence 4 points for circularity; the calcified segment is < 1.5 mm, therefore 3 points for thickness; the calcification is continuous, thus 4 points for morphology. Given a cumulative score of 11, image A depicts a medial IAC. (**B**) Calcification spans between 90-270° of arterial circumference, hence 3 points for circularity; the calcified segment is >1.5 mm, therefore 1 point for thickness; the calcification is irregular/patchy, thus points for morphology. Given a cumulative score of 5, image B depicts an intimal IAC.

The patterns of IAC subtypes (intimal or medial calcifications) were classified according to a previous calcification scoring method [10, 30]. The CT imaging-based approach for IAC classification is well-supported by established histology-validation studies [7-10]. These studies have demonstrated a high degree of concordance between CT imaging findings and histopathological confirmation, supporting the reliability of CT-based methods in identifying IAC subtypes. From Table 1, the characteristic patterns of IAC were scored based on the calcification circularity, thickness, and morphology, and the sum of calcification scores was used to classify predominantly intimal (1-6 points) and predominantly medial calcifications (7-11 points). This classification was based on the artery with the highest and predominant calcification score. Figure 1 depicts medial and intimal IAC based on CT classification.

**Table 1:** Assessment of IAC.

(Kockelkoren’s method)[6]				
**Circularity** **Grade 0 No calcification** **1 Dot(s)** ** 2 <90 degrees** ** 3 90-270 degrees** ** 4 270-360 degrees**	*Thickness* Grade 0 No calcification 1 Thick ≥ 1.5 mm 3 Thin < 1.5 mm	*Morphology* Grade 0 Indistinguishable 1 Irregular/patchy 4 Continuous
**(Babiarz’s method)[29]**				
***Extent*** **Grade 0 No calcification** **1 Dot of calcification** **2 Crescentic <90 degrees** **3 Calcification spanning 90-270** **4 Calcification spanning 270-360 degrees of the vascular wall circumference**		*Thickness* Grade 0 No calcification 1 Calcification 1 mm thick 2 Calcification 2 mm thick, thin continuous, or thick discontinuous 3 Calcification 3 mm thick, or thick continuous 4 Calcification >3 mm thick		

Kockelkoren’s method: Characteristic intimal and medial IAC patterns are based on cumulative scores from circularity, thickness, and morphology. Babiarz methods: Assessment of IAC severity is based on cumulative scores based on extent and thickness.

### Protocols of renal Doppler ultrasound

A Philips ultrasound system (Epiq7, Philips, Seattle, USA) equipped with a 1.0- to 5.0-MHz curvilinear array probe was used to examine the kidney by an experienced sonographer blind to all the clinical data of the participants. The ultrasound modality was equipped with a duplex function for evaluating colour-coded blood flow (preferably with additional options for visualising low-velocity blood flow) and recording the blood flow spectrum or other parameters. The scanning parameters for colour Doppler and spectral modes were optimised using the user-defined preset specifically tailored for the renal Doppler application on the Epiq7 Philips ultrasound equipment.

Renovascular haemodynamic assessment was based on arterial resistance using the blood flow resistive indices (RI). This metric was selected based on a recent review of the literature that revealed that the RI is influenced by pulse pressure, renovascular resistance and arterial compliance [31]. Supported by substantial evidence, the review further highlighted that renal artery RI should be considered a reliable marker of systemic cardiovascular risk beyond its prognostic relevance for renal impairment. In this current study, the measurement of RI was taken from two points: the proximal renal artery and the intrarenal or interlobar artery. The RI for each arterial segment was based on peak systolic velocity (PSV) and end-diastolic velocity (EDV). PSV, EDV, and RI values from the left and right kidneys were calculated and averaged for analysis. RI measured at the proximal renal artery is designated renal RI, whereas the RI measured at the intrarenal or interlobar artery is designated interlobar RI. Our study consistently uses the term “renovascular haemodynamics" to refer to the overall dynamics of blood flow within the renal vasculature. The RI is employed as a primary metric to quantify renovascular resistance, providing a transparent and standardised measure for analysis.

### Statistical analyses and power calculations

SPSS (IBM SPSS Statistics 29.0.1.0, Chicago, U.S.A.) was used for all data analysis. Descriptive analyses described demographic characteristics, vascular risk factors, and imaging and laboratory findings. Continuous variables were represented either as mean (with standard deviation) or median (with interquartile range). Clinical variables and haemodynamic parameters were compared between patients with predominantly medial and intimal IAC using the independent samples t-test or Mann-Whitney U-test. Categorical variables were represented as frequencies in percentages and compared using the Pearson chi-square test.

A multinomial logistic regression was used to ascertain the influences between renovascular haemodynamics and the different categories of renal functioning.

Spearman's linear correlation analysis, as well as univariate and multivariate linear regression models, were fitted to explore the independent association between renovascular haemodynamic metrics and IAC. Here, renal RI and interlobar RI were predicting variables against the outcomes of IAC load and IAC severity. In the multivariate models, covariate adjustments were made for age, gender, stroke, hypertension, diabetes, hyper-lipidaemia, coronary heart disease, history of stroke, use of medication, and current smoking and drinking statuses. An ordinal regression model further elucidated the precise order of the associations to ascertain how renovascular haemodynamics related to increasing loads of IAC. A moderation analysis was performed to evaluate the potential interaction effects or medication-induced modifications in renal and cerebral haemodynamics.

**Table 2:** Baseline characteristics.

	Total population	Predominantly Intimal IAC (n=20)	Predominantly Medial IAC (n=49)	P value
Age (x̅±SD)	69.42 ± 13.02	61.55 ± 11.81	72.63 ± 12.19	<0.001
BMI (x̅±SD)	23.43 ± 3.50	23.83 ± 3.86	23.27 ± 3.38	0.551
Systolic BP (x̅±SD)	155.32 ± 25.45	150.85 ± 23.69	157.14 ± 26.14	0.355
Diastolic BP (x̅±SD)	89.16 ± 14.76	89.70 ± 16.99	88.94 ± 13.93	0.848
HbA1c [M(IQR)]	6.30 (5.75-7.40)	6.15 (5.55-7.00)	6.30 (5.80-7.60)	0.435
Diabetes (n, %)	19 (24.70%)	2 (10.0%)	17 (34.69%)	0.037
TCH [M(IQR)]	4.60 (3.92-5.94)	5.75 (4.26-6.30)	4.42 (3.57-5.26)	0.012
TG [M(IQR)]	1.42 (1.07-2.11)	1.37 (1.07-3.41)	1.47 (1.05-2.00)	0.721
HDL-C [M(IQR)]	1.06 (0.91-1.29)	1.03 (0.92-1.31)	1.07 (0.90-1.29)	0.706
LDL-C (x̅±SD)	3.17 ± 0.93	3.54 ± 0.77	3.02 ± 0.96	0.035
Hcy [M(IQR)]	14.20 (10.83-17.42)	13.19 (9.16-16.93)	14.30 (11.35-17.95)	0.606
D-Dimer [M(IQR)]	0.63 (0.25-1.12)	0.35 (0.20-0.96)	0.80 (0.35-1.19)	0.034
FIB [M(IQR)]	3.30 (2.70-3.95)	3.15 (2.32-3.40)	3.50 (2.80-4.20)	0.024
SCr [M(IQR)]	89.33 (66.4-122.19)	71.29 (58.30-93.53)	94.81 (71.93-135.63)	0.003
eGFR (x̅±SD)	78.46 ± 42.37	104.16 ± 32.16	67.96 ± 41.79	<0.001
IAC count [M(IQR)]	4 (3-5.5)	4 (1.25-5)	5 (4-6)	0.051
IAC Severity [M(IQR)]	9 (6-10)	5 (5-6)	10 (8-10)	<0.001
IAC Load (x̅±SD)	33.96 ± 15.17	19.60 ± 10.61	39.82 ± 12.67	<0.001
Interlobar PSV [M(IQR)]	25.5 (21.0-25.5)	29.5 (25-35.5)	24 (19.25-30.75)	0.023
Interlobar EDV [M(IQR)]	8.80 (7.04-10.71)	11.62 (9.08-13.66)	7.89 (6.67-9.25)	<0.001
Renal PSV [M(IQR)]	78.0 (62.0-95.0)	71.75 (61.25-96.75)	81.50 (63.75-95.0)	0.552
Renal EDV [M(IQR)]	21.24 (16.2-24.9)	24 (19.99-32.00)	20.46 (14.80-23.88)	<0.001
Interlobar RI (x̅±SD)	0.66 ± 0.08	0.61 ± 0.06	0.68 ± 0.07	<0.001
Renal RI (x̅±SD)	0.73 ± 0.07	0.67 ± 0.04	0.75 ± 0.06	<0.001

x̅ and M represent mean and median respectively; SD, standard deviation; IQR, interquartile range; TG, triglyceride; TCH, total cholesterol (mmol/L); HDL-C, high-density lipoprotein cholesterol (mmol/L); LDL-C, low-density lipoprotein cholesterol (mmol/L); Hcy, homocysteine; FIB, fibrinogen; SCr, serum creatinine (µmol/L); eGFR, estimated glomerular filtration rate (ml/min/1.73m^2^); IAC, intracranial arterial calcification; IAC_SB, IAC score burden; PSV, peak systolic velocity (cm/s); RI, resistive index; ILA, interlobar artery; RA, renal artery, homocysteine (µmol/L), and fibrinogen (g/L). The p-values for parametric continuous variables, expressed as mean ± standard deviation (x̅±SD), were obtained using t-tests. For non-parametric continuous variables, shown as a median and interquartile range [M(IQR)], p-values were derived from Mann-Whitney U-tests. Categorical variables, presented as counts and percentages (n, %), had their p-values determined using Pearson’s Chi-Square tests.

Power calculations were performed using G*Power 3.1, utilising a moderate correlational effect size of 0.37 based on the outcomes from previous studies [32-34]. This calculation revealed that 84 participants would be needed to attain statistical significance with 95% power and an α-level of 0.05. However, we collected data from 77 participants, with 69 identified as positive cases. Despite the reduced sample size, recalculations (using G*Power 3.1) revealed a robust power of 90%.

Given the small sample size (77), the significant predicting variables, following the univariate regressions, were standardised and converted as z-scores to prevent the exaggeration of odd ratios in multivariate analyses. This approach enabled us to prevent bias and enhance the interpretability of regression results. Standardised z-score $=\frac{\left(X-\mu \right)}{\sigma }$, where *X* is the original value, *μ* is the mean of the dataset, and *σ* is the standard deviation. No missing variables were noted in the data. A two-sided p-value <0.05 was considered statistically significant.

## RESULTS

### Participant’s characteristics

Among the 77 participants, 69 (89.6%) with a mean age of 69.42 ± 13.02 years exhibited intracranial arterial calcification, with 20 (29%) predominantly intimal and 49 (71%) predominantly medial IAC. On average, each participant had four counts of calcification lesions in various arterial beds of the brain. Of these individuals, 46 (59.7%) were male, 57 (74%) had ischaemic strokes. Additionally, 56 (72.7%) were hypertensive, with 48 (85.7% of 56) taking antihypertensive medications. Nineteen participants (24.7%) had diabetes, and all were on antidiabetic medicines. Thirty-six (52.2%) had hyperlipidaemia, with thirty-five of them on lipid-lowering drugs. Nine (11.7%) had coronary heart disease, and 12 (15.6%) had a history of stroke, with 11 of these individuals taking antiplatelet or anticoagulant medications. Furthermore, 18 (23.4%) were current smokers, and 7 (9.1%) consumed alcohol. For those with IAC, the average eGFR score was 78.46 ± 42.37 ml/min/1.73m². Of these, 26 (37.7%) had an eGFR below 60 ml/min/1.73m², 19 (27.5%) had values between 60-90 ml/min/1.73m², and 24 (34.8%) had scores above 90 ml/min/1.73m². Four (5.8%) patients with IAC had end-stage renal disease with eGFR <7 ml/min/1.73m², respectively. Table 2 provides a summary of the clinical variables measured in this population.

**Table 3:** Distribution of IAC and eGFR between patients stratified by RI ≥ 0.70 and RI < 0.70.

Parameter	Renal RI ≥ 0.70 (n= 44)	Renal RI < 0.70 (n= 25)	P value	Interlobar RI ≥ 0.70 (n= 26)	Interlobar RI < 0.70 (n= 43)	P value
Patients with IAC (n = 69)	44 (63.8%)	25 (36.2%)	< .001	26 (37.7%)	43 (62.3%)	< 0.001
IAC load (mean rank)	43.4	20.22	< .001	44.83	29.06	< 0.001
Patient with eGFR < 60 ml/min/1.73 m²	24 (54.5%)	2 (8%)	< .001	61.5% (16/26)	10 (23.3%)	0.004

This table compares the distribution of IAC and eGFR among patients stratified by renal and interlobar resistive index (RI) values: ≥ 0.70 and RI < 0.70. Renal RI: Among patients with a renal RI ≥ 0.70 (n=44), 63.8% had IAC, and the mean rank of IAC load was significantly higher compared to those with a renal RI < 0.70 (n=25), where 36.2% had IAC (p < .001). A significantly higher proportion of patients with a renal RI ≥ 0.70 had an eGFR < 60 ml/min/1.73 m² (54.5%) compared to those with a renal RI < 0.70 (8%) (p < .001). Interlobar RI: For patients with an interlobar RI ≥ 0.70 (n=26), 37.7% had IAC, with a significantly higher mean rank of IAC load compared to those with an interlobar RI < 0.70 (n=43), where 62.3% had IAC (p < .001). A significantly greater percentage of patients with an interlobar RI ≥ 0.70 had an eGFR < 60 ml/min/1.73 m² (61.5%) compared to those with an interlobar RI < 0.70 (23.3%) (p = 0.004).

### Association between renovascular haemodynamics, eGFR, and IAC

As shon in Table 3, most of those with higher renal or interlobar RI measures presented with reduced renal functions. A multinomial logistic regression analysis demonstrated that lower eGFR scores, particularly those <60 ml/min/1.73m², were independently associated with higher renal RI (Table 4). This association was evident in both the unadjusted model [OR, 3.70 (95% CI: 1.76 - 7.80, p<.001)] and the adjusted model [OR, 3.45 (95% CI: 1.38 - 8.59, p = 0.008)].

Again, Table 3 shows that patients with increased renovascular resistance (RI ≥ 0.70) exhibit a greater IAC load compared to those with lower renovascular resistance (RI < 0.70). The correlation analysis (Table 5) shows that there are statistically significant and positive correlations between renovascular resistance metrics and IAC load, IAC severity, and IAC subtype. There was, however, no statistically significant correlation between renovascular resistance metrics and IAC count.

From the regression models (Table 6), models 1 and 2 show an independent association between renal RI and the IAC load [crude OR = 2.81 (95% CI: 1.75 - 4.52); adjusted OR = 1.88 (95% CI: 1.06 - 3.35). Furthermore, ordinal regression analysis in model 3 demonstrated that higher renal RI levels are linked to greater IAC severity burden [OR = 2.15 (95% CI: 1.29 - 3.59). Given the standardised nature of RI values, the multivariate linear regression outcome in model 2 suggests that for every 0.08 standard deviation increase in renal RI, the odds of having a higher IAC load increase by 1.88 times, regardless of underlying risk factors such as age, gender, stroke, hypertension, diabetes, hyperlipidaemia, coronary heart disease, history of stroke, and use of medication, as well as current smoking and drinking statuses.

**Table 4:** Multinomial logistic regression of renovascular resistance on eGFR.

eGFR	Unadjusted Model OR (95% CI, p-value)	Adjusted model OR (95% CI, p-value)
>90 ml/min/1.73m^2^	Ref.	Ref.
60-90 ml/min/1.73m^2^	1.01 (0.52-1.98, p=.967)	0.91 (0.32-2.21, p=.827)
<60 ml/min/1.73m^2^	3.70 (1.76-7.805, p<.001)	3.45 (1.38-8.59, p=.008)

This table illustrates that increased renovascular resistance is significantly linked to severe renal impairment, especially in patients with an eGFR below 60 ml/min/1.73m², compared to those with an eGFR above 90 ml/min/1.73m². Unadjusted model: unadjusted multinomial logistic regression Adjusted model: accounted for risk factors such as age, gender, stroke, hypertension, diabetes, hyperlipemia, coronary heart disease, history of stroke, use of medication, current smoking and drinking statuses.

In the same analyses, linear regression analysis indicated that interlobar RI was associated with IAC load in model 1 [OR = 2.30 (95% CI: 1.40 - 3.78). Ordinal regression analysis in model 3 also demonstrated that higher interlobar RI levels were linked to greater IAC severity, with an OR of 2.32 (95% CI: 1.31 - 4.09). However, in model 2, the multivariate regression analysis suggested that the association between interlobar RI and IAC load is not statistically significant when adjusted for vascular risk factors, despite a positive odds ratio [adjusted OR, 1.55 (95% CI: 0.86 - 2.77, p = 0.141)]. These findings suggest that renal RI may serve as a more robust and reliable predictor of IAC load.

**Table 5:** Correlation between renovascular flow resistance and IAC.

	Spearman's rho	P value	95% Confidence Intervals	
			Lower	Upper
**RI (Interlobar artery)**				
IAC count	.105	.393	-.143	.339
IAC load	.421	<.001	.198	.603
IAC severity	.417	<.001	.193	.600
IAC subtype	.427	<.001	.205	.607
**RI (Renal artery)**				
IAC count	.018	.882	-.226	.260
IAC load	.422	<.001	.199	.603
IAC severity	.354	.003	.121	.550
IAC subtype	.595	<.001	.411	.732

This table presents Spearman's rho correlation coefficients between RI values in both interlobar and renal arteries and various measures of IAC, including count, load, severity, and subtype (n =69). Significant positive correlations were observed for IAC load, severity, and subtype with RI in both interlobar and renal arteries, indicating that increased renovascular resistance is associated with these IAC characteristics.

### Clinical and haemodynamic comparisons between medial and intimal IAC

**Figure 2.**
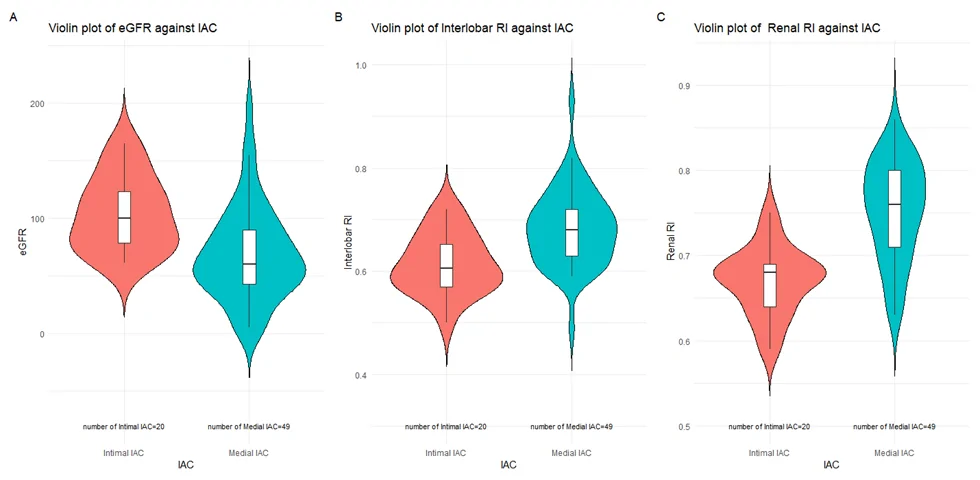
**Violin Plots of Renal Function and Haemodynamics in IAC Patients**. This figure presents the distribution of eGFR scores and RI values for renal and interlobar arteries among 20 patients with predominantly intimal IAC and 49 with predominantly medial IAC. (**A**) Patients with medial IAC exhibit significantly lower eGFR values, indicating more severe renal dysfunction compared to those with intimal IAC. (**B**) Medial IAC is associated with higher RI values in the renal arteries, suggesting increased renovascular resistance. (**C**) Medial IAC is linked to higher RI values in the interlobar arteries, further indicating elevated vascular resistance in these patients.

The Table 2 presents the inferential results from independent t-tests with Cohen's d-effect sizes and Mann-Whitney U-tests, highlighting significant differences between patients with predominantly medial and intimal intracranial arterial calcification (IAC). Patients with predominantly medial IAC were significantly older than those with predominantly intimal IAC (72.63 vs 61.55 years, d = 0.917, p<0.001). Additionally, these patients exhibited lower levels of kidney function, as shown by lower eGFR levels (67.96 vs 104.16 ml/min/1.73m², d = 0.921, p<0.001), as illustrated in Figure 2 (A). Notably, all four patients with end-stage renal disease exhibited medial IAC. In contrast, patients with predominantly intimal IAC had significantly higher LDL-C levels compared to those with medial IAC (3.54 vs 3.02 mmol/L, d = 0.570, p = 0.035).

Mann-Whitney U tests revealed that the severity of IAC was significantly higher in patients with predominantly medial calcifications compared to those with predominantly intimal calcifications (6.0 vs 4.5, U=109, p<0.001). Again, those with medial IAC, when compared to those with intimal IAC, recorded higher levels of D-dimer (0.80 vs 0.35, U = 330, p = 0.034), higher fibrinogen (3.50 vs 3.15 g/L, U=319.50, p = 0.24), higher serum creatinine (94.81 vs 71.29 µmol/L, U=262, p = 0.003). Meanwhile, individuals with predominantly intimal IAC, when compared to those with medial IAC, recorded significantly higher levels of total cholesterol (5.75 vs 4.42 mmol/L, U= 300, p = 0.012).

Notably, the two groups had no significant differences in BMI, gender, systolic and diastolic blood pressure, triglycerides, HDL-C, and homocysteine levels. Both groups recorded relatively similar counts of calcifications across various intracranial beds.

As shown in Table 2, independent t-test analyses revealed that patients with predominantly medial IAC had significantly higher levels of renovascular blood flow resistance compared to those with predominantly intimal IAC. These outcomes are illustrated in Figure 2 (B & C) by the higher resistive indices observed in the renal arteries (0.75 vs 0.67, d = 0.980, p<0.001) and the interlobar arteries (0.68 vs 0.61, d = 0.962, p<0.001).

**Table 6:** Linear and ordinal regression between resistive indices and IAC scores.

Predicting Variable	Model 1 OR (95% CI, p-value)	Model 2 OR (95% CI, p-value)	Model 3 OR (95% CI, p-value)
Renal RI	2.81 (1.75-4.52, p<.001)	1.88 (1.06-3.35, p=.032)	2.15 (1.29-3.59, p=.004)
Interlobar RI	2.30 (1.40-3.78, p<.001)	1.55 (0.86-2.77, p=.141)	2.32 (1.31-4.09, p=.004)

The table presents the robustness of renal and interlobar RI, assessed via regression models in 69 patients with IAC. Model 1 is the Unadjusted linear regression model depicting the association between the resistive index and the IAC load. Model 2 adjusted for age, gender, stroke, hypertension, diabetes, hyperlipemia, coronary heart disease, history of stroke, use of medication, and current smoking and drinking statuses in the linear regression. Model 3 is the ordinal regression outcome revealing that increasing resistive index is associated with a higher IAC severity burden.

### Medication-induced interactions in renal/cerebral haemodynamics

The moderation analysis was performed using the PROCESS macro for SPSS (Model 1), with IAC load as the outcome variable (Y), renal artery RI as the predictor (X), and medications as the moderators (W). From Table 7, the analysis showed a significant direct effect of renal artery RI on total IAC load. However, the main effect of antihypertensive, antidiabetic, and lipid-lowering medications and their interactions were not significant. There were minimal or no changes in R² due to the interactions (ΔR² = 0.02, 0.00, and 0,03, respectively).

**Table 7:** Medication-induced interactions in renal/cerebral haemodynamics.

Medication Type	Direct Effect of Renal Artery RI (b, p-value)	Main Effect of Medication (b, p-value)	Interaction Effect (b)	R² Change	p-value (Interaction)
Antihypertensive	18.67, p = 0.001	10.25, p = 0.063	-9.45	0.02	0.187
Antidiabetic	12.78, p = 0.002	4.45, p = 0.600	0.16	0.00	0.987
Lipid-Lowering	12.88, p = 0.001	-20.91, p = 0.138	23.12	0.03	0.145

The table presents the results of moderation analyses examining the interaction between renal artery RI and medication use on IAC load (n = 69). The direct effect of renal artery RI indicates a significant positive association with IAC load across all medication types. The main effect of each medication type on IAC load was not statistically significant. Interaction effects assess whether medication use modifies the relationship between renal artery RI and total IAC load, with none of the interactions reaching statistical significance. R² Change reflects the proportion of variance explained by the interaction term. The p-value for the interaction effect indicates the statistical significance of the moderation.

## DISCUSSION

**Figure 3.**
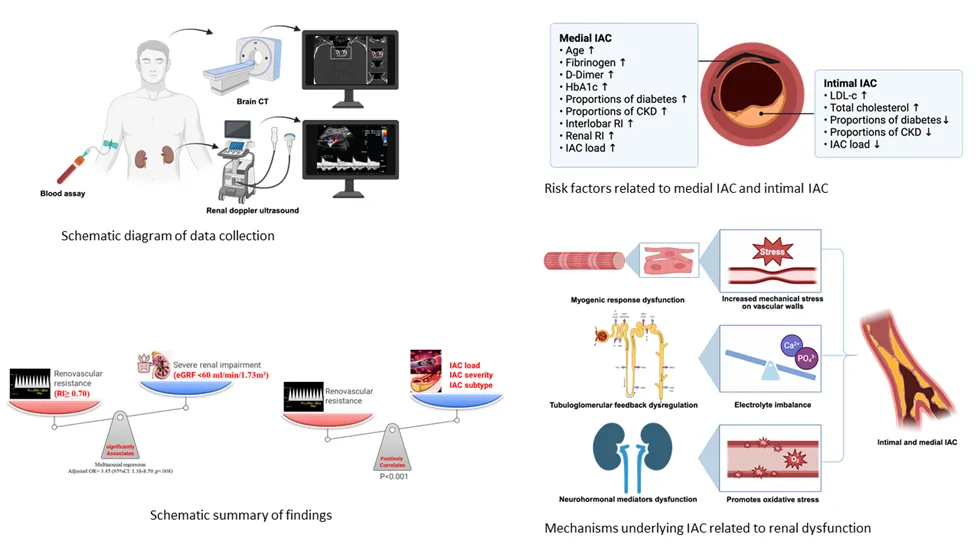
**Schematic summary**. This figure summarises the investigation, key findings and proposed pathophysiological mechanisms. This diagram was created using BioRender (https://BioRender.com).

This study looked at how renal function and renovascular haemodynamic metrics affect the load and severity of IAC and contribute to the clinical distinctiveness between intimal and medial IAC subtypes. Renovascular haemodynamics entailed renovascular blood flow resistance metrics measured by RI in the renal and interlobar arteries. In this study, increases in renovascular resistance to blood flow, reliably indicated by RI, were independently associated with higher loads of IAC. Higher levels of renovascular blood flow resistance in renal and interlobar arteries are associated with reduced renal functions. Compared to patients with predominantly intimal IAC, patients with predominantly medial IAC had higher levels of renovascular blood flow resistance with associated impaired kidney functions, as well as higher loads and severe calcifications. Figure 3 depicts the schematic summary of the entire investigation, key findings, and the proposed pathophysiological mechanisms.

This current study observed an 89.6% prevalence of IAC among the 77 participants, with higher proportions noted in patients with predominantly medial calcification. This result is closely consistent with our previous clinical study that reported 85.27% IAC prevalence among 516 patients [25]. Goldsmith and colleagues, in a longitudinal study with a 16-year mean follow-up, also reported a surge in vascular calcification from 38% to 92% among 38 patients with impaired renal functions [33]. This current study revealed that, on average, patients with IAC have mildly reduced renal function with an average eGFR score of 78.46 ± 42.37 ml/min/1.73m². The findings showed that about 37.7% of patients with IAC will likely have poor renal function with an eGFR below 60 ml/min/1.73m². The findings also indicate that reduced renal function, especially when eGFR is below 60 ml/min/1.73m², is strongly associated with severe burdens of IAC beyond vascular risk factors such as age, gender, stroke, hypertension, diabetes, hyperlipidaemia, coronary heart disease, history of stroke, use of medication, current smoking, and drinking statuses. These findings are consistent not only with our previous clinical study [25] but also with Sedaghat et al.’s population-based study that showed that lower eGFR was associated with higher volumes of calcification in intracranial carotid arteries among individuals with a mean age of 69.5 ± 6.7 years [32]. The current study significantly offers new contributions to the existing body of knowledge by being the first study to elucidate the impact of renovascular haemodynamics on the burden of IAC.

Notably, the results revealed that 63.8% (44 out of 69) of patients with IAC exhibited increased renovascular resistance in the renal arteries, and these patients presented with higher loads of IAC. More than half (54.5%) of these patients had an eGFR of less than 60 ml/min/1.73 m². Following examination of the interlobar arteries, increased renovascular resistance was observed in only 37.7% of patients with IAC, although a significant portion (61.5%) also had eGFR < 60 ml/min/1.73 m²). The current results underscore a strong relationship between renovascular haemodynamics, renal function, and IAC. This study further reinforces this evidence by demonstrating that patients with higher levels of renovascular blood flow resistance are over three times more likely to have impaired renal function (eGFR < 60 ml/min/1.73 m²) compared to those with normal kidney function (Table 4).

Moreover, the current findings suggest that the medial calcification pattern is the predominant subtype of IAC, occurring in 71% of patients with calcification. In attempts to describe the underlying mechanisms of cerebrovascular calcification, Voelkl and his team reported that end-stage kidney disease patients had a notable prevalence of medial calcification [35]. They found the same pattern of medial calcification predominance even in adolescents on dialysis who didn't have any of the usual risk factors for atherosclerosis. The current study adds to this body of literature by highlighting significant differences in the clinical characteristics and risk profiles of patients with predominantly medial versus intimal IAC. Patients with medial IAC tend to be older and exhibit lower kidney function, as indicated by reduced eGFR, suggesting a potential link between renal impairment and medial calcification. This finding aligns with recent studies that associate ageing and renal dysfunction with vascular calcification, emphasising the role of systemic factors in medial IAC development [25]. In contrast, patients with intimal IAC show higher levels of low-density lipoprotein cholesterol, which is consistent with the established relationship between lipid metabolism and atherosclerotic changes in the intima [36, 37]. The increased severity of IAC loads in medial calcifications and elevated D-dimer, fibrinogen, and serum creatinine levels underscores a systemic inflammatory and thrombotic milieu associated with medial calcification [38, 39].

Furthermore, this current study found that the different patterns of IAC may be associated with distinct haemodynamic profiles, potentially influencing clinical outcomes. This current study supports the evidence that the mechanisms and risk factors for arterial media and intima calcifications differ fundamentally from those driving atherosclerotic vulnerable intimal lesions. Several interconnected mechanisms related to renal function and the intrarenal autoregulatory processes are considered. Fluctuations in calcium and phosphate metabolism from renal impairment usually result in abnormal deposition of minerals, particularly in the medial layer of arterial walls [25, 40]. Medial calcification is often accompanied by increased expression of proinflammatory markers, indicating a link between local vascular inflammation and calcification [24, 25]. Again, a decreased renal function can lead to increased oxidative stress. This oxidative stress is known to promote the trans-differentiation of vascular smooth muscle cells into osteoblast-like cells, extracellular matrix remodelling, and progressive dysregulated mineral metabolism (e.g., elevated phosphate and calcium) [39]. Molecular pathways include the downregulation of calcification inhibitors (e.g., pyrophosphate), upregulation of bone-related proteins, and involvement of matrix metalloproteinases that modulate matrix biology and inflammation [41]. These processes predominantly predispose the medial layer of blood vessels to calcifications that exacerbate vascular stiffness and resistance rather than luminal obstruction [42, 43]. Consistent with existing literature [39, 44], our study found that common risk factors, including ageing, diabetes, and severe renal impairment, were more prevalent among patients with predominantly medial IAC.

In contrast, intimal calcification is less influenced by these intrarenal autoregulatory processes. Intimal IAC is shown to be more closely associated with atherosclerotic processes, which involve lipid accumulation, endothelial dysfunction, immune cell infiltration and plaque formation [36, 37]. Low-density lipoproteins penetrate the intima, undergo oxidation, and trigger innate and adaptive immune responses, promoting foam cell formation and plaque progression [44, 45]. Calcification in plaques is often patchy and linked to necrotic core formation, contributing to plaque instability or vulnerability to rupture [45]. Therefore, the unique metabolic and mineral deregulatory conditions induced by decreased renal function predominantly influence medial calcification, with minimal impact on intimal calcification. This link underscores the higher prevalence and increased renovascular resistance noted among patients with predominantly medial IAC. The clinical distinction is crucial for understanding the pathophysiology of IAC in patients with renal impairment.

The findings from this study on the cerebro-renovascular link align with recent investigations into arteriosclerotic disease, underscoring the interdependence of vascular beds. The observed association between renal haemodynamic alterations and IAC severity suggests that perturbations in one arterial territory may propagate to distant sites through shared haemodynamic, metabolic, and neurohormonal pathways [42, 46]. This bidirectional relationship is particularly evident in populations with chronic kidney disease, where uremic toxins and dysfunctional mineral metabolism concurrently drive both renal arteriolar calcification and cerebral arterial disease [28, 34, 47, 48]. The clinical ramifications extend beyond cerebrovascular health, as demonstrated by parallel findings in coronary and peripheral circulations where medial calcification correlates with adverse outcomes, including resistant hypertension and tissue ischaemia [13, 49]. Of particular clinical relevance is the potential mechanistic overlap between renal-derived factors influencing cerebral arterial stiffness and the subsequent development of neurocognitive impairment [50]. These observations collectively emphasise the need for integrated vascular risk management strategies that address renal and cerebrovascular health in tandem. This consideration should be emphasised, particularly in high-risk populations with concurrent metabolic and hypertensive disorders. Our study highlights the importance of renal haemodynamics in the context of intracranial arterial calcification, underscoring the need for comprehensive management strategies to mitigate the risk of these adverse outcomes.

The strengths of this study are underscored in the clinical significance of renovascular haemodynamics, particularly the resistive index, in assessing the load and severity of IAC. The positive correlations between RI in both renal and interlobar arteries with various IAC metrics, such as IAC load and severity, reinforce the potential of RI as not just a diagnostic marker for renal impairment but also a predictive marker for IAC. Independent of common vascular risk factors, the renal RI could serve as a robust predictor of IAC, offering clinicians a valuable tool for risk stratification. Clinically, these insights could inform more targeted screening and management strategies for patients at risk of IAC, potentially improving outcomes by identifying those who may benefit from more intensive monitoring or therapeutic interventions. The standardised nature of RI values further enhances its utility, as demonstrated by the finding that a 0.08 standard deviation increase in renal RI significantly raises the odds of a higher IAC load and that this is profound among patients with an eGFR score <60 ml/min/1.73m². It is important to note that while interlobar RI also shows an association with IAC load and severity, its predictive power diminishes when adjusted for vascular risk factors. This finding further indicates that renal RI, measured at the level of the proximal renal artery, could be a more reliable indicator of IAC load compared to the intrarenal or interlobar artery RI.

The study employed rigorous methods to mitigate potential biases. Data were standardised for regression models to prevent overestimating odds ratios, ensuring that the statistical analyses accurately reflected the relationship between variables. Furthermore, the study carefully accounted for various risk factors, such as age, gender, stroke, hypertension, diabetes, hyperlipidaemia, coronary heart disease, history of stroke, use of medication, and current smoking and drinking statuses, to minimise confounding effects and isolate the specific impact of renovascular haemodynamics on IAC. The analyses further suggested that while renal artery RI consistently predicts IAC load, the use of antihypertensive, antidiabetic, and lipid-lowering medications does not significantly moderate this relationship in our sample. This outcome indicates that the effect of renal artery RI on IAC load is mainly independent of these medications, at least within the scope of our study. However, it is essential to consider that the absence of significant interactions does not preclude the possibility of medication-induced modifications in other haemodynamic parameters or different contexts.

The diagnostic relevance of these findings lies in the potential for renovascular haemodynamic non-invasive measurements to enhance the prediction and risk assessments of IAC, thereby facilitating earlier and more effective clinical decision-making. To translate this into clinical practice, we propose incorporating renal RI measurements into routine evaluations, especially for patients with renal impairment, hypertension, diabetes, or dyslipidaemia. Using Doppler ultrasound, clinicians can identify patients at higher risk for IAC based on specific renal RI thresholds (RI>0.7 indicating higher renovascular resistance). This approach will enable targeted monitoring and preventive strategies. Additionally, tracking renal RI over time could provide insights into the progression of arterial calcification and the effectiveness of interventions. The findings from this study justify the need to develop multidisciplinary clinical protocols that integrate the clinical management of renal and cerebrovascular health. Such protocols could facilitate the implementation of targeted screening and therapy for IAC in high-risk populations with compromised renal functions.

Although this study provides valuable insights into the relationship between renovascular haemodynamics, renal function, and IAC, several limitations should be acknowledged. The relatively small sample size of 77 Chinese participants may limit the generalisability and statistical power of the findings to broader populations. Our study's initial power calculations indicated that a sample size of 84 participants was necessary to achieve 95% power, assuming a moderate correlational effect size of 0.37 and an α-level of 0.05. However, we collected data from 77 participants, with 69 identified as positive cases. Despite the reduced sample size, recalculations (G*Power 3.1) revealed that the study maintains a robust power of 90%. This high-power level suggests that our research is still well-equipped to detect meaningful associations, even with fewer participants than originally planned. The observed significant correlational effect sizes ranged from 0.35 to 0.59, indicating a spectrum of moderate to strong associations between renovascular haemodynamic metrics and IAC parameters (Table 5). These findings underscore the sensitivity of our study design in capturing these relationships despite the sample size constraints. While the reduced sample size may limit the generalisability of our findings, particularly when stratifying the 69 positive participants into subtypes, the maintained power of 90% provides confidence in the robustness of the detected associations. This power reinforces the validity of our results, offering a solid foundation for subsequent investigations.

This limited sample reflects a broader issue in clinical practice, where comprehensive brain vascular assessments are not routinely performed for patients with renal impairment despite the potential cerebrovascular consequences. This gap suggests a need for more proactive diagnostic strategies to ensure that cerebrovascular risks are adequately assessed and managed in this vulnerable population. Moreover, the cross-sectional design of this study limits our ability to infer causality between renal impairment and IAC. Additionally, our study cohort consists entirely of Chinese patients, which provides a high degree of internal consistency and minimises confounding variables related to genetic and environmental diversity. This homogeneity strengthens the internal validity of our findings. However, this demographic specificity also presents a limitation in terms of external validity. The findings may not be directly generalisable to other ethnic groups due to potential genetic, physiological, and lifestyle differences that can influence renovascular and intracranial dynamics. Therefore, while our results offer valuable insights into the studied population, caution should be exercised when extrapolating these findings to broader, multi-ethnic populations. Future longitudinal studies with diverse cohorts are needed to validate the predictive value of renal RI for IAC progression.

Again, using CT-based morphological features to differentiate intimal from medial IAC subtypes offers a non-invasive and practical approach to clinical assessments. Nonetheless, histopathological validation remains the gold standard, providing definitive insights into arterial wall composition and changes. We therefore recommend that future research continue to explore ways to enhance the accuracy of imaging-based differentiation. Integrating histopathological features with advanced imaging modalities or machine learning techniques to refine IAC subtype classification could improve the effectiveness of the quantitative assessment of IAC.

In conclusion, our findings suggest that renovascular haemodynamics significantly influence the IAC load and severity, with exacerbated effects observed in cases of poor renal function. Additionally, renal impairment and renovascular resistance have a more pronounced impact on medial IAC compared to intimal IAC, highlighting the distinct characteristics of IAC patterns. The exacerbated effects of renal impairment and increased renovascular resistance on IAC underline the need for integrated clinical management strategies in both kidney and cerebral large artery diseases. Therefore, coupling CT and ultrasound imaging to enhance the precision detection, classification, and management of IAC subtypes among patients with compromised kidney functions will yield optimal clinical impact.

## Data Availability

The data supporting this study's findings are not publicly available because they contain information that could compromise the privacy of research participants. However, upon reasonable request, the corresponding author, [X.C], at fiona.chen@cpce-polyu.edu.hk, can make these data available.
